# Abnormal Weight Loss in an Adolescent Female With Down Syndrome

**DOI:** 10.7759/cureus.26822

**Published:** 2022-07-13

**Authors:** Monica Garcia, Florentina Litra

**Affiliations:** 1 Pediatrics, University of Florida, Pensacola, USA; 2 Department of Pediatrics, Ascension Sacred Heart, Pensacola, USA

**Keywords:** weight loss adolescent female, down syndrome, developmental regression, abnormal weight loss, down syndrome disintegrative disorder

## Abstract

Down syndrome is the most common chromosomal abnormality identified at birth. These individuals will have multiple comorbid illnesses that require complex medical care throughout their lives. In recent years, specific patterns of regression have been detected in these individuals; most notably, developmental regression with language, behavior, and cognitive skills that they had previously acquired, which now affects both the quality of life and autonomy. These development regression patterns are referred to as Down syndrome disintegrative disorder.

The case that we are reporting occurred in a 17-year-old female presenting with significant weight loss, altered mental status, and loss of functional skills over a period of one month. Co-occurring hypothyroidism and hyperthyroidism symptoms exacerbation may have triggered this patient’s regression. Our case highlights the importance of conducting a thorough investigation for life-threatening and non-life-threatening illnesses that can present with similar symptoms, in order to make the correct diagnosis of Down syndrome disintegrative disorder and find appropriate therapies and future care.

## Introduction

Individuals with Down syndrome, at times, have multiple comorbid illnesses that are present at birth or develop later on in life. This makes for complex medical care throughout their lives, involving multiple specialists to care for them appropriately. The case that we are reporting occurred in a 17-year-old female presenting with significant weight loss, altered mental status, and loss of functional skills over a period of one month. Co-occurring hypothyroidism and hyperthyroidism symptoms exacerbation may have triggered this patient’s developmental regression. Our case highlights the importance of conducting a thorough investigation for life-threatening and non-life-threatening illnesses that can present with similar symptoms, in order to make the correct diagnosis of recently established Down syndrome disintegrative disorder to help individuals and their families find appropriate or future therapies.

## Case presentation

A 17-year-old female, with a history of Down syndrome, congenital hypothyroidism, patent foramen ovale, celiac disease, bilateral cataracts, and bilateral conductive hearing loss was admitted to the inpatient pediatric medical unit after an approximate 8 kg (18 pounds) weight loss over a month, with associated altered mental status.

Initially, the patient's mother noticed persistent complaints of abdominal pain followed by weight loss. She was initially seen at a local outside emergency department where she was diagnosed with a urinary tract infection and had a normal thyroid profile (Table 1) showing thyroid-stimulating hormone (TSH) of 0.02 mcIU/mL and free T4 of 4.12 ng/dL. After about a week of oral antibiotics and continued weight loss, the patient's mother started to note some loss of interest in eating, interacting with family and friends, and in activities she previously enjoyed. The patient’s mother sought further evaluation with the labs seen in Table 1 by the patient's pediatrician, who repeated the thyroid profile, showing a TSH of <0.02 mcIU/mL (range 0.47-3.41 mcIU/mL) and free T4 of 4 ng/dL (range 0.58- 1.64 ng/dL), thyroid peroxidase antibody >900 IU/mL (range <35 IU/mL), thyroid stimulating immunoglobulin antibodies of 178 IU/L (range < 0.54 IU/L), negative hepatitis panel, and negative HIV polymerase chain reaction (PCR), all seen in Table 1. In the light of this apparent hyperthyroidism, the patient’s pediatrician consulted with the primary pediatric endocrinologist (whom she follows for her congenital hypothyroidism), who recommended levothyroxine be discontinued and an ultrasound of the abdomen, ultrasound of the thyroid, and MRI of the pituitary gland be performed. Both ultrasounds were within normal limits, and MRI had not yet been done. Over the next seven to 10 days, the patient frequented her pediatrician’s office with continued weight loss and abdominal pain, new-onset confusion, decreased concentration, night sweats, subtle tremors, and dysphagia.

**Table 1:** Initial outside diagnostic laboratory results TSH: thyroid-stimulating hormone; PCR: polymerase chain reaction; PCP: primary care physician

Diagnostic lab	Patient Value	Range
Emergency department labs		
TSH	0.02 mcIU/mL	0.47-3.41 mcIU/mL
Free T4	4.12 ng/dL	0.58- 1.64 ng/dL
PCP office labs		
TSH	<0.02 mcIU/mL	0.47-3.41 mcIU/mL
Free T4	4 ng/dL	0.58- 1.64 ng/dL
Thyroid peroxidase antibody	>900 IU/mL	<35 IU/mL
Thyroid-stimulating immunoglobulin antibodies	178 IU/L	< 0.54 IU/L
Hepatitis PCR panel (A,B,C)	Negative	Positive or Negative
HIV 1 & 2 antigen PCR	Negative	Positive or Negative

At the time of admission to the inpatient pediatric unit, the patient's weight was 45.6 kg (14.8% percentile according to the CDC’s growth chart for children with Down syndrome). Per the mother’s report, the patient’s previous weight was around 53 kg, which placed her in the 34% percentile on the growth chart. On initial examination, significant Down syndrome phenotypic features, such as flat facial profile, epicanthic fold, short stature, brachydactyly, sandal gap, and global hypotonia, were noted, along with a flat affect, non-verbal, brisk bilateral knee reflexes but no focal deficits. Initial blood work in the hospital (Table 2) was significant for WBC 12.2 K/uL (range 4.0-11.0 K/uL), neutrophil 78.7%, absolute neutrophil 9.6 K/uL (range 1.6-8.5 K/uL), complete metabolic panel with electrolytes (calcium, magnesium, phosphate), sedimentation rate, amylase, and lipase were within normal limits. Liver function testing showed normal range for AST and ALT and elevations in the total bilirubin level at 2 mg/dL (range 0.1-0.8 mg/dL), uric acid at 7 mg/dL (range 2.5-6.5 mg/dL), and LDH at 122 Intl Units/L (125-220 Intl Units/L). Urinalysis results showed WBC 5/HPF (range 0-10/HPF), ketones 5 mg/dL, positive leukocyte esterase, and negative nitrates. A repeat thyroid panel in the pediatric unit showed a TSH of 20.21 mcIU/mL (range 0.47-3.41 mcIU/mL) and free T4 0.05 ng/dL (range 0.58-1.64 ng/dL).

**Table 2:** Abnormal hospital laboratory results SH: thyroid-stimulating hormone; PCR: polymerase chain reaction

Diagnostic Lab	Patient Value	Range
WBC	12.2 K/uL	4.0-11.0 K/uL
Neutrophil	78.7%	25%-68%
Absolute neutrophil	9.6 K/uL	1.6-8.5 K/uL
Total bilirubin	2 mg/dL	0.1-0.8 mg/dL
Prealbumin	13.2 gm/dL	3.5-5.0 gm/dL
Uric acid	7 mg/dL	2.5-6.5 mg/dL
LDH	122 Intl Units/L	125-220 Intl Units/L
TSH	20.21 mcIU/mL	0.47-3.41 mcIU/mL
Free T4	0.05 ng/dL	0.58- 1.64 ng/dL
Folate	5.4 ng/mL	7.0-31.4 ng/mL
Vitamin B12	>2,000 pg/mL	213-816 pg/mL
Urinalysis		
WBC	5/HPF	0-10/HPF
Ketones	Trace	Positive, Negative, Trace
Leukocyte esterase	Positive	Positive, Negative, Trace
Nitrates	Negative	Positive, Negative, Trace

Differential diagnosis

Our patient’s initial presentation of weight loss, mental status changes, and developmental regressions broadened our differentials to include endocrine abnormalities such as hyperthyroidism, hypothyroidism, pituitary adenoma, anemia, and vitamin deficiency. Gastrointestinal-related diagnoses included esophagitis, gastritis, Crohn’s disease, and peptic ulcer. Neurological and psychiatric diagnoses included depression, physical/sexual assault, subclinical seizures, space-occupying brain lesions, and Down syndrome disintegrative disorder.

Diagnosis 

The final diagnosis of Down syndrome disintegrative disorder was made after an extensive workup that included multiple laboratory tests and diagnostics. Our primary goal for this patient was to prevent any further weight loss and helping the patient and her family to return as close to her baseline as possible. However, due to the broad differential that the patient’s admission started with, it was imperative that we rule out any life-threatening diagnosis.

After initial labs resulted in a significantly abnormal thyroid profile, the endocrinologist recommended restarting Levothyroxine and rechecking her thyroid function in two weeks. They also recommended checking cortisol and adrenocorticotropic hormone (ACTH) levels (normal range). Although there was a strong suspicion that patients’ symptoms were due to stopping the thyroid medication, further workup to rule out other diagnoses from our differential list was continued. Further investigation was conducted to rule out any vitamin deficiencies that could cause weight loss and lack of interest and energy, which can be seen in Table 2. Vitamin D 25-OH was within the normal range and folate was 5.4 ng/mL (7.0-31.4 ng/mL range). Hemoglobin and hematocrit were within the normal range, ruling out macrocytic anemia, and vitamin B12 was > 2,000 pg/mL (range 213-816 pg/mL). After speaking with the patient's mother, it was discovered that she was taking a B12 supplement every other day, which would cause such elevated levels. However, her symptoms were not thought to be related to toxicity; it is not possible to have vitamin B12 toxicity since it is a safe water-soluble vitamin that is not stored in the body in excess and excreted in the urine [1]. After restarting the patient's Levothyroxine, the pediatric neurologist was consulted for recommendations on workup for this patient’s mental status change. An EEG was placed for 24 hours to rule out subclinical seizures or encephalopathy, which was normal. MRI brain with and without contrast with attention to the pituitary gland was also within normal limits with no lesions, hypodensities, or hyperdensities to indicate other pathology. Our next step was to consult with a pediatric gastroenterologist for a workup of the patient's weight loss, abdominal pain, and food aversion. Initial recommendations were to start with empiric proton pump inhibitors for possible esophagitis/gastritis, with which daily Lansoprazole 15 mg was initiated. It was also recommended to check gamma-glutamyl transferase (GGT), tissue transglutaminase immunoglobulin A (IgA) and IgG, serum IgA to rule out an exacerbation of her celiac disease, and calprotectin and pre-albumin to rule out the possibility of Crohn’s disease. These labs were all within normal limits except for pre-albumin at 13.2 gm/dL (range 3.5-5.0 gm/dL), which can be seen in Table 2. After blood work, a repeat US abdomen was recommended due to continued abdominal pain, which was also normal. Another diagnostic recommendation was a barium swallow study due to food aversion and dysphagia that showed no anatomical abnormalities or delay in gastric emptying. Since the initial gastrointestinal workup was normal, an esophagogastroduodenoscopy was performed and per the GI team, it demonstrated diffuse erythema with slight nodularity in the stomach, with normal esophagus and duodenum. Continued use of Lansoprazole was recommended; however, the GI team did not suspect that this was the sole reason for her significant weight loss. After ruling out life-threatening neurologic diseases, the psychiatric team was consulted to evaluate for a major depressive disorder that would explain her weight loss, lack of interest, non-verbal behavior, decreased energy, or decrease in basic life skills. The psychiatric team recommended starting the patient on mirtazapine 7.5 mg at bedtime. Further investigation was conducted to rule out any vitamin deficiencies that could cause weight loss, lack of interest, and energy. After this extensive investigation through peer-reviewed journals, research and case studies, and laboratory and diagnostic studies, the concluding diagnosis of Down syndrome disintegrative disorder due to immune dysregulation was made.

Management

For this condition, the management was based on alleviating symptoms and optimizing their health and wellness. In this instance, it began with restarting Levothyroxine, which was previously discontinued by the patient’s endocrinologist due to low levels of TSH and free T4, causing significant symptoms. Titration of this medication would be done on an outpatient basis with endocrinology.

Apart from optimizing her thyroid hormone, management of weight loss and food aversion was key to achieving appropriate nutritional status. Her nutritional status was initially managed with fluids and then the Ensure (Abbott Nutrition, Chicago, Illinois) and Jevity (Abbott Nutrition) nutritional drinks via nasogastric (NG) and oral feeds were added on. These forms of feeding were started during her inpatient stay and weaned as the patient started to eat regular food by mouth on her own with encouragement from her family. To help mitigate any further abdominal pain due to gastritis, a proton pump inhibitor (lansoprazole) was started and continued after discharge. To also mitigate any worsening depressive symptoms and sleep disturbances, mirtazapine was started and continued after discharge.

Patient course 

Throughout the eight-day course of admission in the inpatient pediatric unit, the patient's nutritional status was optimized. At first, due to the severity of her weight loss, food aversion, and selective mutism, as previously discussed, she was started on intravenous fluids and encouraged to eat and drink anything by mouth. Due to this weight loss, the inpatient dietitian was consulted to help optimize the nutritional status with an appropriate number of calories.

After the initial EEG and MRI study was completed, she was started on nasogastric (NG) continuous feeds at night with Jevity 1.5 at a rate of 45 ml/hr from 6:00 pm to 6:00 am. During the daytime, she was encouraged to drink a minimum of two Ensure nutritional drinks along with any snacks and meals. It was recommended by the inpatient dietitian that she consumes at least 75% of her nutritional needs by mouth. Throughout her stay, her oral intake improved enough that she was able to be saline locked by the third day, and her NG feeds were stopped a few days prior to her discharge. With encouragement from her parents and sibling, the patient was starting to eat more food and drink juice on her own. Due to the severity of her food aversion and decreased nutritional intake, the patient had changes in her bowel movements, which required the addition of probiotics to help mitigate the constipation that she had prior and the loose stools she was currently having from the NG feeds. Due to the fact that it had been almost a month of minimal food intake, we monitored her vitals, bowel movements, urine output, and electrolyte levels for signs of refeeding syndrome.

During her stay, due to the variety of symptoms and regression of daily living skills, speech therapy, physical therapy, and occupational therapy were provided to the patient daily to help motivate and encourage her to start building her skills once again. As previously discussed, psychiatry was also consulted for signs of major depressive disorder, and the patient was started on nightly mirtazapine, which helped improve her sleep and energy levels.

On the day of discharge, the patient had a final weight of 46.3 kg, an increase of 700 grams in a little over a week, about 87.7 grams on average increase per day. The mother and patient were encouraged to keep on increasing her food intake and drinking Ensure one to two times a day. It was also recommended to continue the use of lansoprazole and mirtazapine, with follow-up to pediatric endocrinology, pediatric gastroenterology, and psychiatry. At the end of her inpatient stay, the patient had not completely recovered to her previous development status or mental status but she was on the road to recovery with time.

## Discussion

Down syndrome is the most common chromosomal abnormality that is identified at birth, and it is the most common syndromic cause of intellectual disability [2]. The diagnosis is often made during prenatal screening in the first and second trimesters with maternal serum testing for pregnancy-associated plasma protein-A (PAPP-A) and total human chorionic gonadotropin (HCG) and quadruple testing with alpha-fetoprotein (AFP), Estriol, HCG, and Inhibin A [3]. The classical phenotypic clinical features that these individuals present range from up-slanting palpebral fissures to epicanthic folds, brachycephaly, and flat facial profile and/or global hypotonia. Approximately half of the individuals with Down syndrome present with congenital heart disease, most commonly with complete atrioventricular septal defects and ventricular septal defects. As for gastrointestinal disorders, there are strong associations with celiac disease and Hirschsprung’s disease. A small percentage, about 5%, have duodenal atresia or annular pancreas. Another prevailing abnormality in these individuals is hypothyroidism and type 1 diabetes [2]. Many present with comorbid psychiatric and behavioral disorders such as autism, ADHD, and/or aggressive behavior. As these individuals age, dementia and Alzheimer’s disease become prevalent around the age of 35 [2].

After this extensive investigation through peer-reviewed journals, research and case studies, and laboratory and diagnostic studies, the concluding diagnosis of Down syndrome disintegrative disorder due to immune dysregulation was made. In recent years, there have been multiple reports of specific patterns of regression in individuals with Down syndrome, especially developmental regression with language, behavior, and cognitive skills that had previously been acquired and affect both the quality of life and autonomy of these individuals. This is now being referred to as Down syndrome disintegrative disorder [4] or unexplained regression in Down syndrome [5].

Most of the individuals diagnosed with this disorder have multiple features in multiple categories and present around the age of 15-16-year-old with an equal female to male ratio of 1:1.3 [5]. In the past, clinicians have tried to gather information to establish core features and identified possible triggers to form diagnostic criteria that include regression in adaptive functioning, cognitive-executive functions, motor control, and common behavioral and mental health features that are detailed in Table 3 [5].

**Table 3:** Core features of Down syndrome disintegrative disorder ADL: activities of daily living; PTSD: post-traumatic stress disorder; EEG: electroencephalogram

Adaptive Functioning		
	Social skills:	Withdrawal, avoidance, isolation; time spent alone
	Functional ADLs:	Loss of acquired skills; dependent
	Speech:	Reduced, infrequent; whisper, monosyllabic, or mute
Cognitive-Executive Function		
	Attention	Atypical, odd: gaze aversion, poor eye contact, or impaired ocular control
	Functional skills:	Loss, confused, disorganized; unable to function at school/work
	Procedural memory	Less able to perform or performs with the assistance needed regarding ADL routines or favorite activities
	Learning memory	Diminished working memory; not processing or learning
	Planning, organizing	Not goal-directed, disorganized
	Declarative memory	Forgetful and confused with regard to people, places, and events
Motor Control		
	Initiation-motivation	Abulia, avolition, mutism
	Stereotyped movements	Tics, stereotypes
	Catatonia	
	Extrapyramidal	Bradykinesia, freezing, cogwheel rigidity, tremor
Behavior Features		
	Internalizing	Apathy, withdrawal, mood, stereotype, self-injurious behavior
	Externalizing	Hyperactivity, irritable, disruptive, agitated
Mental Health Features		
	Mood, emotion, self-regulation	Depression, compulsions, psychosis, PTSD, anxiety, panic, autism spectrum disorder/Down syndrome disintegrative disorder
	Sleep disturbance	Insomnia, circadian shift
	Transition/change	causing emotional distress in the past 1 year
	Appetite	Anorexia, weight loss
	Incontinence	Urine, feces
	Trauma/loss/grief	Causing emotional distress in the past 1 year
	Puberty	Causing emotional distress in the past 1 year
	Illness/hospitalization	Causing emotional distress in the past 1 year
	Sleep apnea, seizures	Evidence on polysomnogram, EEG
	Other inflammatory, autoimmune condition	
	Systemic illness	Pain, surgery
	Autonomic	Syncope, pallor, swelling
	Vision, hearing	Acute loss or deterioration

Across the multiple studies focusing on Down syndrome disintegrative disorder, five possible causes have been proposed, which include “early changes associated with Alzheimer's disease, disruption in routines or loss of support associated with the transition to adulthood, disruption in self-identity, the inherent risk for catatonia, and autoimmunity” [5].

However, in order to confidently say that an individual has Down syndrome disintegrative disorder after identifying core features and life stressors/triggers, it is key to perform a thorough medical evaluation to identify any other possible abnormalities that may explain such symptoms or can be co-occurring with this regression. In both Santoro et al. and Rosso et al., there is a proposed tiered evaluation that includes both radiologic and diagnostic studies as seen in Table 4.

**Table 4:** Diagnostic workup for regression TSH: thyroid-stimulating hormone; Ig: immunoglobulin; PANDAS: pediatric autoimmune neuropsychiatric disorders associated with streptococcal infections; EEG: electroencephalogram; ESR: erythrocyte sedimentation rate; CRP: C-reactive protein; RPR: rapid plasma reagin

Tier	Diagnosis	Evaluation
Tier 1	Thyroid disorders: hyperthyroidism, hypothyroidism	Thyroid function: TSH, free T2, thyroid peroxidase antibodies, thyroglobulin antibodies
	Electrolyte disturbances, infections	CBC, electrolytes (Ca, Mg, Po, Na, K)
	Vitamin deficiency	Folate, vitamin B12, 25-OH vitamin D
	Celiac disease	Anti-tTg, total IgA
	Obstructive sleep apnea	Polysomnography
	Hearing loss	Hearing test
	Vision loss	Vision screen
	Constipation	X-ray abdomen
	Depression/anxiety	Depression and life stressors screen
	Other neurologic disorders	MRI brain
	Other psychiatric disorder	Psychiatric referral
Tier 2	PANDAS	Antistreptolysin O
	Seizure disorder	EEG
	Other immunologic disorders	Antinuclear antibodies, ESR, CRP
	Syphilis, HIV	RPR, HIV serology

In the study by Santora et al., 35 individuals were identified from published and unpublished studies, 23 individuals had abnormalities found within tier one and within tier two of the diagnostic workup, and five individuals had been found to have abnormalities. Although these individuals had co-occurring conditions, it was not likely that was the sole explanation for the regression disorder but rather an exacerbation of it [5]. In Rosso et al, a meta-analysis of three different historical reports was performed in which language regression and mood symptoms were the most likely reported complaints at 58% and 42%, respectively. Based on these historical reports, Rosso et al. performed further evaluation in which two cohorts in which 20% (n=4 of 20) of individuals had MRI brain studies done, showing abnormalities like dementia with hippocampal atrophy [4] while in the other 80%, no abnormalities were able to be detected. As for that 20%, they proposed that it could be associated with the early onset of Alzheimer’s.

Identification of this disorder is key since acute regression can last for about six months and then turn to a more chronic regression course in a way that does not allow for recovery of any of their previous developmental skills. In pediatric patients, it was found that in two studies, about 58% of the patients diagnosed with Down syndrome disintegrative disorder reportedly had partial or total recovery with 35% stabilizing and 7.5% experiencing worsening symptoms [4]. 

It has been proposed that the diagnosis should be based on the presence and/or absence of the 28 core features in the questionnaire in Table 3, from which this scoring system was proposed. Diagnosis of Down syndrome disintegrative disorder was said to be unlikely if the patients scored 0-3, a possible diagnosis with a score between 4 and 9, probably this diagnosis if between 9 and 15, and highly likely this diagnosis if it was 16 or greater. This questionnaire and scoring system would allow physicians to refer to appropriate specialists with experience in helping individuals with this regressive disorder and help unify the clinical research that is out there [5].

Since causes for this disorder have not been proven, it becomes difficult to treat individuals even if abnormalities were found on medication evaluations. The use of low-dose psychotropic medications, electroconvulsive therapy, and even immunotherapy has been proposed [5].

## Conclusions

During her stay, due to the variety of symptoms and regression of daily living skills, speech therapy, physical therapy, and occupational therapy were provided to the patient daily to help motivate and encourage her to start building her skills once again. As previously discussed, psychiatry was also consulted for signs of major depressive disorder, and the patient was started on nightly mirtazapine, which helped improve her sleep and energy levels.

At the end of her extensive diagnostic and laboratory evaluation, it was concluded that changing her medications and restarting high school was a large enough trigger for her to present with this clinical regression. Since this condition is one of regression from baseline inpatient physical therapy, occupational therapy and speech therapy were consulted and daily therapy sessions were provided while inpatient. Combining all of these therapies was key to helping achieve near-complete recovery for individuals with Down syndrome disintegrative disorder. Although the use of low-dose psychotropic medications, electroconvulsive therapy, and immunotherapy has been proposed while working with a multidisciplinary team. Therapy, medications, and close follow-up are key to recovery.
